# Sustainable and Flexible Surface-Enhanced Raman Scattering Transducer: Gold Nanoparticle-Bacterial Cellulose Composite for Pesticide Monitoring in Agrifood Systems

**DOI:** 10.3390/bios15020069

**Published:** 2025-01-23

**Authors:** Daniela Lospinoso, Adriano Colombelli, Sudipto Pal, Pasquale Cretì, Maria Concetta Martucci, Gabriele Giancane, Antonio Licciulli, Roberto Rella, Maria Grazia Manera

**Affiliations:** 1 Istituto per la Microelettronica e i Microsistemi Unit of Lecce CNR-IMM, Campus Ecotekne Via Monteroni, 73100 Lecce, Italy; adriano.colombelli@cnr.it (A.C.); pasquale.creti@cnr.it (P.C.); mariaconcetta.martucci@cnr.it (M.C.M.); roberto.rella@cnr.it (R.R.); 2 Department of Engineering for Innovation, Campus Ecotekne, University of Salento, Via Monteroni, 73100 Lecce, Italy; sudipto.pal@unisalento.it (S.P.); antonio.licciulli@unisalento.it (A.L.); 3 Department of Cultural Heritage, University of Salento, Via D. Birago, 73100 Lecce, Italy; gabriele.giancane@unisalento.it

**Keywords:** nano-fibrillated bacterial cellulose, AuNPs, wastepaper, plasmonic biosensors, SERS assay

## Abstract

Functionalized plasmonic nanostructure platforms are widely used for developing optical biosensors and SERS assays. In this work, we present a low-cost and scalable surface-enhanced Raman scattering (SERS) system based on an innovative optical transducer comprising gold nanoparticles (AuNPs) embedded in nano-fibrillated bacterial cellulose (BC). The AuNPs@BC composite leverages the unique nanofibrillar architecture of bacterial cellulose, which provides a high surface area, flexibility, and uniform nanoparticle distribution, enabling the formation of numerous electromagnetic “hot spots”. This structure excites localized surface plasmon resonance (LSPR), as demonstrated by a bulk sensitivity of 72 nm/RIU, and supports enhanced Raman signal amplification. The eco-friendly and disposable AuNPs@BC platform was tested for agrifood applications, focusing on the detection of thiram pesticide. The system achieved a detection limit of 0.24 ppm (1 µM), meeting the sensitivity requirements for regulatory compliance in food safety. A strong linear correlation (R^2^ ≈ 0.99) was observed between the SERS peak intensity at 1370 cm^−1^ and thiram concentrations, underscoring its potential for quantitative analysis. The combination of high sensitivity, reproducibility, and environmental sustainability makes the AuNPs@BC platform a promising solution for developing cost-effective, flexible, and portable sensors for pesticide monitoring and other biosensing applications.

## 1. Introduction

Recent advancements in nanomaterial science and engineering have significantly expanded the applications of surface-enhanced Raman scattering (SERS) for detecting analyte traces in various fields, including environmental monitoring, biosensing, catalysis, and food safety. SERS technology leverages the localized surface plasmon resonance (LSPR) properties of metallic nanostructures, particularly noble metals like gold, silver, and copper, to amplify the Raman signals of adsorbed molecules. Gold nanoparticles (AuNPs) are widely favored for SERS applications due to their excellent chemical stability, biocompatibility, and tunable LSPR properties, making them highly effective at amplifying weak Raman signals from target analytes. The use of AuNPs as SERS substrates has been extensively studied focusing on the development of novel nanocomposites to improve the sensitivity and reproducibility of detection systems [1].

Supporting AuNPs on bacterial cellulose (BC) nanofibrils represents a promising direction for creating environment-friendly, flexible, and efficient SERS substrates. BC is a natural biopolymer produced by bacteria, such as *Gluconacetobacter xylinum*, and has gained increasing attention due to its remarkable properties, including high purity, high crystallinity, non-toxicity, super flexibility, biodegradability, and higher mechanical strength [2,3].

BC consists of a highly crystalline nanofibrils structure, which offers a large surface area and porosity, making it an ideal scaffold for embedding nanomaterials like AuNPs [4]. The BC morphology and the presence of high concentration of hydroxy groups make BC superhydrophilic, allowing strong chemical bonding of the positively charged Au^3+^ ions to the cellulose fiber surfaces and subsequent reduction, resulting in uniform distribution of the AuNPs, which is critical for generating “hot spots”—regions of intense electromagnetic field enhancement that are essential for strong SERS signal amplification [5]. The integration of AuNPs into BC xerogel not only enhances the SERS performance but also maintains the biocompatibility and flexibility of the substrate, making AuNPs@BC composites highly attractive for practical sensing applications, in particular for food safety monitoring.

Food safety is a critical issue, and the detection of pesticide residues on fruits, vegetables, and other agricultural products is of paramount importance.

Thiram is an organosulfer pesticide and fungicide widely used but not admitted in European community for its toxicity to humans. It is extensively used in agricultural practices, particularly in fruits and vegetables, as a fungicide in countries outside the European Union. Overuse of thiram in agricultural products may lead to diseases and various health issues in humans and livestock due to its potent accumulation effect. Hence, the detection of thiram residues on the surface of agricultural products holds significant practical importance [6].

In the U.S., the EPA regulates the maximum amount of thiram residues, referred to as pesticide tolerances. Based on risk assessments, the specific tolerance limits vary depending on the type of food. For example, the tolerance for thiram residues on apples is set at 5 ppm [7]. In the European food safety authority (EFSA) review, the limit of quantification (LOQ) for thiram residues was set at 0.01 ppm for all commodities [8]. Any residue above this limit is considered non-compliant with the regulations, making it impossible to sell or import products containing amounts of thiram above this threshold.

Traditional methods for detecting thiram, such as high-performance liquid chromatography (HPLC) and gas chromatography–mass spectrometry (GC-MS), are highly sensitive but time-consuming, expensive, and require complex sample preparation [9]. In contrast, SERS offers a rapid, sensitive, and cost-effective alternative for detecting thiram residues in food, with the potential for on-site analysis [10].

Gold nanoparticles play a pivotal role in SERS-based detection systems, particularly for analytes like thiram. When light interacts with AuNPs, the collective oscillation of conduction electrons on the nanoparticle surface results in a dramatic amplification of the local electromagnetic field, known as the LSPR effect. This phenomenon significantly enhances the Raman scattering of molecules adsorbed on or near the AuNP surface, allowing for the detection of even trace amounts of the analyte [11]. The intensity of the SERS signal goes with the fourth power of the electric field, meaning that even minor improvements in nanoparticle design or distribution can lead to a significant increase in sensitivity [12]. AuNPs exhibit strong LSPR in the visible and near-infrared regions, typically between 500 and 600 nm, depending on their size, shape, and aggregation state. This tunable plasmonic property makes AuNPs highly versatile in various SERS detection platforms [13].

In general, the synthesis of AuNP/BC composites is performed following the popularTurkevich method, wherein gold chloride (HAuCl_4_) is reduced by trisodium citrate in the presence of BC nanofibrils [14]. BC serves as both a template and a stabilizing agent for the AuNPs, ensuring their uniform distribution throughout the nanofibrillar matrix. The negatively charged cellulose nanofibrils attract the cationic Au ions, which are then reduced by citrate to form AuNPs directly on the BC surface. This method results in a well-dispersed AuNPs@BC composite with a high density of “hot spots”, enabling enhanced SERS sensitivity for detecting target molecules like thiram. Several studies have demonstrated the effectiveness of AuNPs@BC composites for SERS-based sensing, with detection limits for thiram reaching parts per billion (ppb) levels, making these substrates ideal for real-time monitoring of pesticide residues [14].

The combination of AuNPs and BC offers additional advantages for SERS applications. BC is highly biocompatible and biodegradable, making it an environmentally friendly alternative compared to the synthetic polymers. Additionally, BC’s mechanical flexibility allows for the fabrication of flexible and wearable SERS sensors, which can be easily integrated into various portable sensing devices. The porous structure of BC also facilitates the rapid diffusion of analytes to the AuNP surface, enhancing the sensitivity and response time of the SERS sensor [10]. Moreover, BC’s ability to retain moisture and maintain a stable microenvironment makes it suitable for biosensing applications in humid or aqueous conditions, such as detecting contaminants in food or water samples [4].

In this paper, we report the synthesis of gold nanoparticles deposited onto the nano-fibrillated BC to obtain AuNPs@BC composites by in situ chemical reduction of Au^3+^ ions in an aqueous medium. BC as well as AuNPs@BC composite formation were fully characterized by atomic force microscopy (AFM), optical spectroscopy in the UV-VIS spectral range, and refractometric measurements in a refractive index region suitable for biosensing application. Tests were conducted to put in evidence a suitable surface-enhanced Raman scattering (SERS) assay to thiram pesticide detection.

## 2. Materials and Methods

### 2.1. Chemicals and Reagents

All the chemicals used in the synthesis were of reagent grade. Gold chloride (III) solution (HAuCl_4_, 99.99% trace metals basis, 30 wt% in dilute HCl), and tetramethyl thiuram disulfide (Thiram) 97% powder were purchased from Sigma-Aldrich, Darmstadt, Germany. Sodium citrate tribasic dihydrate (Na_3_C_6_H_5_O_7_·2H_2_O, 99%) was purchased from Alfa-Aesar. Milli-Q grade water was used in all processes.

### 2.2. Synthesis of AuNPs and Bacterial Cellulose (AuNPs@BC) Transducers

Pure bacterial cellulose pellicles from the kombucha strains were prepared following the procedures described in our earlier work [15]. Thin BC hydrogels were cut into small pieces and placed in a high-speed blender to prepare a suspension consisting of the BC nanofibrils. AuNPs@BC suspension was prepared by simultaneously reducing Au (III) ions on the BC nanofibrils by a modified Turkevich method using trisodium citrate as the reducing agent [16]. To prepare this, the fibrillated BC suspension was mixed with a specific amount of water (H_2_O) containing HAuCl_4_·3H_2_O in a Schott Duran borosilicate glass bottle fitted with a polypropylene screw cap. The mixture was vigorously stirred in a water bath maintaining the temperature of about 80 °C using a magnetic stirrer with a hot plate, which allowed the cationic Au ions to stick on the BC nanofibrils. This can be readily visible by observing the light yellowish color of the BC fibrils. At this time the suspension was heated to boiling and a specified amount of 1 wt% trisodium citrate solution was dropwise added to the suspension in stirring condition. After 10 min of reaction, the color of the suspension gradually changed from light pink to pink and finally wine red. The boiling was continued for 30 min and then allowed to stir at room temperature for 2 h to allow the AuNPs to attach to the BC nanofibrils. Thus s gold nanoparticle-BC composite was obtained and denoted as AuNPs@BC. The suspension was centrifuged and washed with deionized water several times to remove any impurities and finally filtered using Whatman 40 filter paper in a Buchner funnel (Sigma-Aldrich, Darmstadt, Germany) and vacuum suction. A uniform film was deposited on the filter paper, which was carefully removed after drying at 70 °C overnight, resulting in a free-standing and flexible AuNPs@BC thin sheet. The film was cut into pieces with desired dimensions and stored at room temperature for further experimentations.

### 2.3. Morphological, Optical and SERS Characterization

The X-ray diffraction (XRD) analysis of AuNPS@BC was conducted using a Rigaku Ultima X-ray diffractometer (Rigaku Europe SE, Neu-Isenburg, Germany) with Cu Kα radiation (λ = 1.5406 Å), operating at 40 kV and 20 mA, with a step size of 0.02°. Morphological characterization of the film was performed using a Zeiss Sigma VP field emission scanning electron microscope (FESEM, Carl Zeiss Microscopy, LLC, White Plains, NY, USA). The morphological and chemical mapping of the samples was carried out with an NT-MDT NTEGRA SPECTRA AFM system (Zelenograd, Russia), which integrates an atomic force microscope (AFM) with a Micro Raman Spectra-C spectrometer (MS 3504i #015022, SOLAR TII, SOL instruments, Augsburg, Germany) and a 1024 × 400 pixel CCD detector (Andor). The laser illumination was provided from above, with the optical path perpendicular to the sample, and signal collection was conducted in reflection mode (backscattered signal collection with an upright configuration). The excitation source was a linearly polarized laser with a wavelength of 632.8 nm (maximum 30 mW), focused using a Mitutoyo M Plan Apo 100× microscope objective (long working distance, numerical aperture 0.7, Mitutoyo Italiana S.r.l., Milano, Italy) positioned above the sample under ambient pressure, temperature, and humidity. The scattered and reflected light was collected by the same objective, spectrally filtered with an edge filter (632.8 nm plus Rayleigh scattering), and directed to a dispersive spectrometer equipped with a 600/600 lines/mm grating. The laser spot position was precisely controlled using a piezo-driven mirror.

A MicroRaman Xplora (Horiba) with a laser at 785 nm (power 0.125 mW·cm^−2^) was used for recording Raman spectra in a different spectral range. MicroRaman Xplora (Horiba Italia S.r.l, Roma, Italy) with a laser at 785 nm (power 0.125 mW·cm^−2^) was used for recording Raman spectra. Laser power was kept under 0.5 mW·cm^−2^ to prevent fluorescence phenomena and the substrate being damaged [17].

SERS experiments were performed on AuNPS@BC substrates functionalized by applying a 5-microliter drop of ethanolic thiram solution at different concentrations and left to air-dry completely.

Spectral acquisitions on SERS substrate by NTEGRA SPECTRA system were performed on ten different points of each thiram-functionalized sample, with an incident laser power of about 1.5 mW (ND filter 1), integration time 100 s, pinhole aperture 50 (corresponding to a monochromator slits size of about 18 µm).

The optical transmittance (400–800 nm spectral range) was measured in air at normal incidence with a Cary 500 UV-vis-NIR double beam Spectrometer (Varian, Palo Alto, CA, USA) and normalized to the corresponding signal of a bare BC without gold nanoparticles.

The sensing properties of AuNPs@BC composites were evaluated by refractometric tests. The refractometric performance of these materials was assessed by impregnating cellulose sheets with test solutions containing increasing concentrations of glycerol, a common agent for tuning refractive index in sensing studies. During the measurements, the plasmonic response was monitored in real time, allowing the evaluation of the sensitivity and responsiveness of the composite materials. The measurements were carried out with a UV-Vis spectrophotometer equipped with a sample holder that allowed the direct immersion of cellulose sheets in glycerol solutions. The glycerol concentration was incrementally increased, and the plasmonic shift was recorded in terms of wavelength shift and intensity variation in the localized surface plasmon resonance (LSPR) absorption peak. The monitoring process was conducted continuously to track the response dynamics as the refractive index of the external environment varied. This methodology ensured a reliable assessment of the plasmonic behavior under different refractive index conditions, which is crucial for sensing applications.

## 3. Results

### 3.1. AuNPs@BC Transducers Synthesis and Characterization

**Figure 1:**
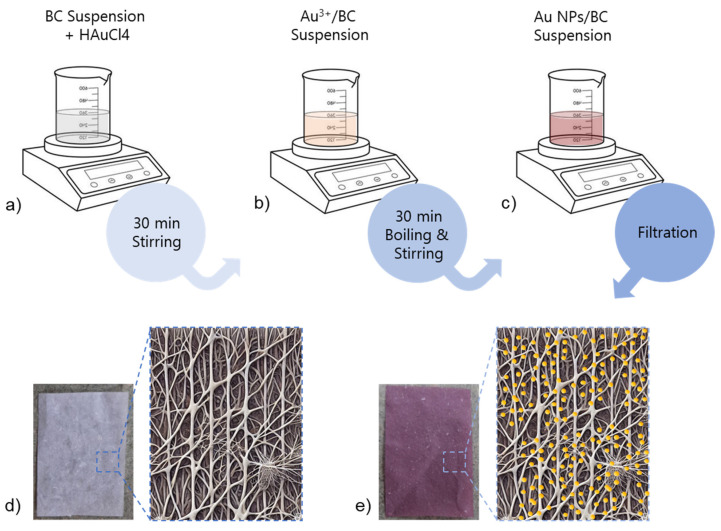
Top: Fabrication process of AuNPs@BC composites involving (**a**) stirring, (**b**) boiling, and (**c**) filtration. Bottom: Photographs of bacterial cellulose (BC) sheets with (**e**) and without (**d**) gold nanoparticles (AuNPs), alongside schematic representations of the nanofibrillar structure of BC and the distribution of AuNPs.

Figure 1 illustrates the phases of the BC@AuNPs film fabrication. The synthesis of AuNP-decorated BC substrates was typically performed using a modified Turkevich method [16], wherein gold chloride (HAuCl_4_) is reduced by trisodium citrate in the presence of BC nanofibrils. BC serves as both a template and stabilizing agent for the AuNPs, ensuring their uniform distribution throughout the nanofibrillar matrix. The negatively charged cellulose nanofibrils attract the cationic Au ions, which are then reduced by citrate to form AuNPs directly on the BC surface. This method results in a well-dispersed AuNPs@BC composite with high-density “hot spots”, enabling enhanced SERS sensitivity for detecting target molecules like thiram. Several studies have demonstrated the effectiveness of AuNPs@BC composites for SERS-based sensing, with detection limits for thiram reaching parts per billion (ppb) levels, making these substrates ideal for real-time monitoring of pesticide residues [14].

**Figure 2:**
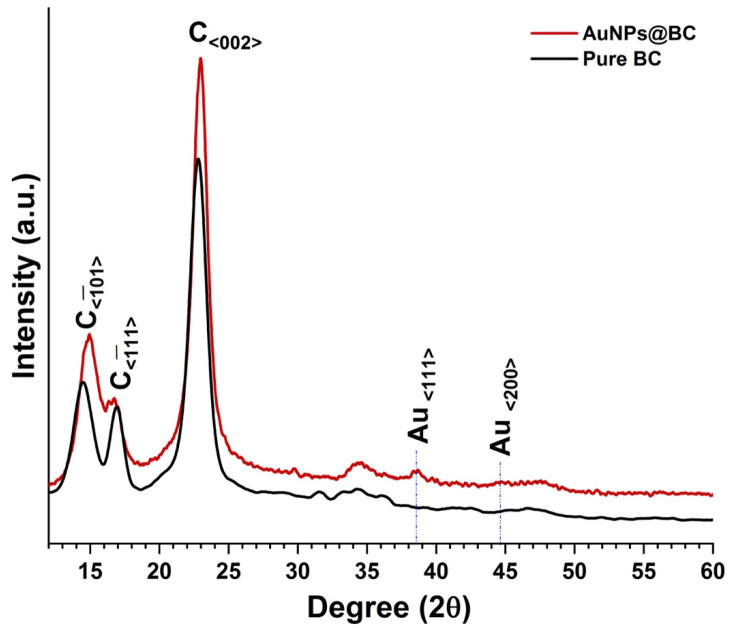
XRD spectra of the AuNPs@BC and pure BC films. Diffraction peaks marked as C corresponds to the nanocrystalline cellulose planes.

Figure 2 shows the XRD spectra of the AuNPs@BC film along with pure BC film as the reference. In both the cases several diffraction peaks are observed. At lower 2θ angles some well-defined broad and intense diffraction peaks are observed that can be indexed (marked as C in Figure 2) as the nanocrystalline cellulose coming from BC [2]. In the case of AuNPs@BC film, two additional small and broad peaks are also visible centered at 2θ values of 38.2° and 44.6° confirming the presence of AuNPs [18] inside the BC matrix. The appearance of the low-intensity diffraction peaks indicates the formation of relatively smaller AuNPs inside the BC matrix.

**Figure 3:**
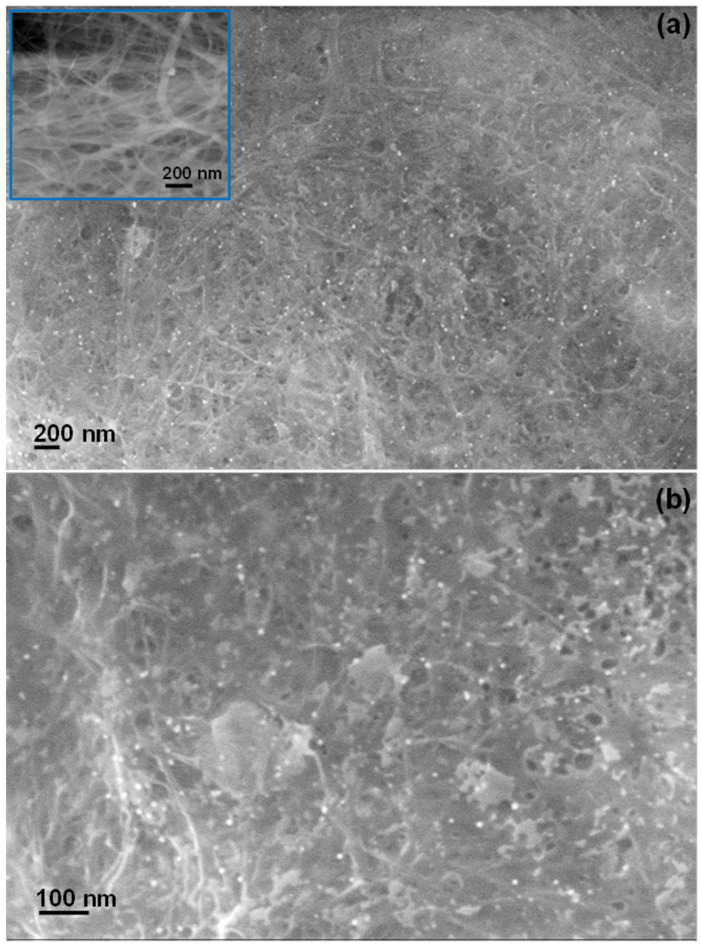
(**a**,**b**) FESEM images of the AuNPs@BC film with different magnifications. Micrograph of pure BC film is shown as inset in (**a**).

FESEM images of the AuNPs@BC film at different magnifications are shown in Figure 3. The inset of Figure 3a shows the SEM image of pure BC film prepared following the same procedure for AuNPs@BC film in the absence of AuNPs. The nanofibrous and porous feature of BC is visible from the figure (inset, Figure 3a) as well as individual cellulose nanofibers with diameters ranging from 20 to 40 nm. AuNPs@BC film shows the homogeneous distribution of the AuNPs (bright spherical particles) inside the cellulose fibrous matrix. Individual AuNPs and the cellulosic nanofibrous network are more clearly visible in Figure 3b. The average size of the AuNPs could be estimated to be about 20–25 nm.

In this study, 2D AuNPs@BC substrates, useful for both nano plasmonic refractive index detection and SERS transducers composed of bacterial cellulose (BC) and AuNPs, were fabricated.

**Figure 4:**
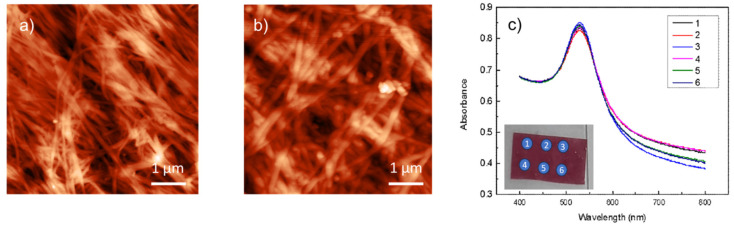
AFM images of bacterial cellulose (BC) films without (**a**) and with gold nanoparticles (AuNPs@BC) (**b**). (**c**) Absorption spectra recorded at different spots on the AuNPs@BC composite film, demonstrating the uniformity of the optical properties across the material.

Figure 4a,b present the AFM topography images in height mode, showing pure bacterial cellulose (BC) alone and BC functionalized with gold nanoparticles (AuNPs). The first image shows the topography of untreated bacterial cellulose revealing the nanoscale fibrillar structure of the bacterial cellulose. The surface appears as a densely intertwined network of fibers with an average diameter of 40 nm, typical of BC. The height variations, depicted in the color scale (ranging from 0 to about 200 nm), highlight the natural roughness and heterogeneity of the cellulose xerogel surface. No distinct additional features are observed beyond the fibrous morphology of the material. The second image (Figure 4b) shows bacterial cellulose modified with gold nanoparticles with a size of approximately 40 nm or less. The underlying cellulose network remains visible, but additional bright spots, representing the gold nanoparticles, are visibly scattered across the surface. These nanoparticles increase the height contrast, appearing as small, elevated features on top of the cellulose fibers. The interaction between the gold nanoparticles and the cellulose leads to distinct topographical modifications, indicating successful functionalization.

Both images, captured at the same scale, emphasize how the presence of AuNPs changes the surface morphology by introducing higher, more localized features compared to the smooth fibril arrangement of pure bare bacterial cellulose.

A typical UV-VIS absorbance spectrum of AuNPs@BC showed a resonant peak in the air at about ~530 nm (Figure 4c). This sharp plasmon resonance at 530 nm suggests that the gold nanoparticles are likely spherical, well-dispersed, and within a narrow size range of approximately 20–30 nm as observed in SEM analyses.

Since aggregation tends to broaden and redshift the plasmonic peak due to particle coupling effects, the sharpness of the peak implies that the nanoparticles are mostly dispersed, rather than forming large aggregates or clusters.

The absorption measurements were performed in different regions of the sample surface, as shown in Figure 4c, in order to test the reproducibility of the typical LSPR absorption peak in different positions to confirm an interesting reproducibility of the optical properties.

A single large area AuNPs@BC SERS active transducer of about 1500 mm^2^ containing a homogeneous distribution of gold nanoparticles can be prepared. Consequently, more reproducible sensing transducers can be selected for specific optical biosensing tests by following the shift in the typical LSPR absorption peak and/or as SERS sensing transducers for agrifood application, as will be shown in the following paragraphs.

### 3.2. LSPR Absorption Peak as a Potential Application in Refractive Index Optical Sensing Measurements

**Figure 5:**
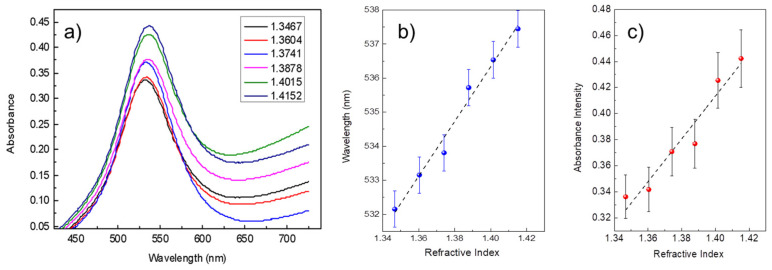
(**a**) UV-Vis absorption spectra of AuNPs@BC composites recorded in media with different refractive indices, showing the plasmonic peak shift. (**b**) Plasmonic peak wavelength and (**c**) intensity variations as a function of the refractive index, demonstrating the bulk sensitivity of the material.

The refractometric measurements reported in Figure 5a provided valuable insights into the sensitivity of the AuNPs@BC composite to variations in the refractive index of the surrounding medium, demonstrating its potential for effective sensing applications. The absorption spectra of Figure 5a show a noticeable red shift, as the refractive index of the external medium increases. This behavior is attributed to the change in the local electromagnetic field around the gold nanoparticles (AuNPs) induced by variations in the surrounding medium. As expected, with the increase in refractive index from 1.3467 to 1.4152, the plasmonic peak shifts to longer wavelengths. This shift indicates the sensitivity of the composite to environmental changes, showcasing its capability to detect even small variations in the surrounding medium.

The graph in Figure 5b, displays the wavelength shift behavior of the plasmonic peak, highlighting a nearly linear relationship between the refractive index and the position of the plasmonic resonance peak. This suggests good bulk sensitivity, as each increment in the refractive index corresponds to a measurable and consistent shift in the plasmonic peak. Such a characteristic is essential for developing reliable optical sensors capable of differentiating between various concentrations of target analytes. Moreover, as shown in Figure 5c, the intensity of the plasmonic peak also shows significant variation with increasing refractive index, demonstrating that the system’s sensitivity is not limited to wavelength shifts but also includes changes in signal amplitude. This dual response, both wavelength and intensity, provides multiple metrics for evaluation, thereby increasing the robustness and reliability of the sensing measurements.

The bulk sensitivity of the AuNPs@BC composite was calculated to further quantify the performance of the material as a plasmonic transducer. The bulk sensitivity, S_bulk_, considering the wavelength shift in response to changes in the refractive index, was found to be S_bulk_ = Δλ/Δn = 72  nm/RIU. This value represents the change in the plasmonic resonance peak position per refractive index unit (RIU) and highlights the ability of the composite to effectively transduce variations in the surrounding environment into a measurable spectral shift. Additionally, the bulk sensitivity with respect to changes in absorbance intensity was calculated as S_bulk_ = ΔI/Δn = 4 RIU^−1^, where ΔI represents the variation in absorbance intensity corresponding to changes in the refractive index. These calculated sensitivities underscore the high efficiency of the composite for refractive index-based sensing, making it a valuable material for applications requiring precise detection of analyte concentrations.

The use of AuNPs embedded within the bacterial cellulose (BC) matrix offers several notable advantages for plasmonic sensing applications. The porous and nanofibrillar structure of BC provides a high surface area, enhancing the interaction between the analyte and the plasmonic “hot spots” generated by the AuNPs. This structural feature ensures uniform solution impregnation and homogeneous interaction throughout the material. The mechanical flexibility of BC enables the fabrication of flexible and adaptable sensing platforms that can be employed in a wide range of environments, including non-planar surfaces, thus enhancing the practical applicability of these sensors for portable and on-site analysis.

### 3.3. SERS Transducers in Agrifood Application

**Figure 6:**
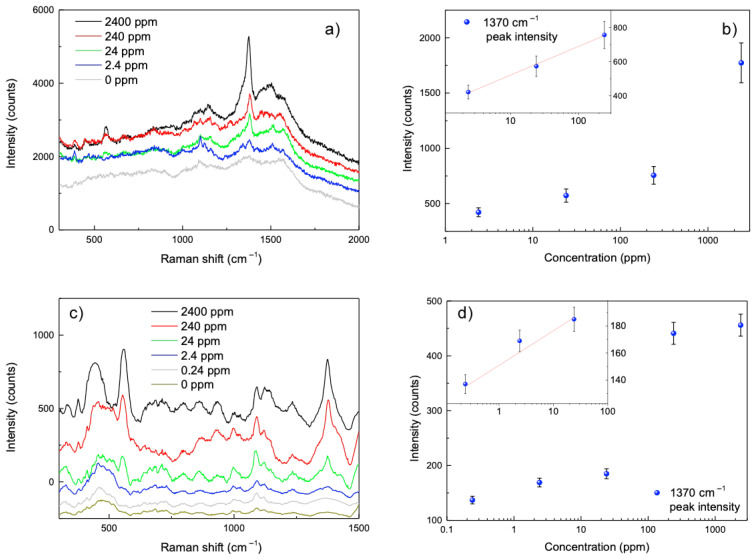
Raman spectra of thiram at increasing concentrations on AuNPs@BC substrates under 633 and 785 nm excitation, respectively (**a**,**c**). The corresponding calibration curves (**b**,**d**) show a linear relationship (insets) between Raman intensity and thiram concentration.

In order to prove the potentialities of the flexible AuNPs@BC SERS sensors in agrifood, the Raman signal from thiram solutions at various ethanol concentrations (from 0.24 to 2400 ppm) drop casted on AuNPs@BC was detected using two distinct measurement apparatuses, as described in Section 2.3. The drop casting method was used instead of dipping, even though the latter should ensure better results [19], because the former is closer to the real sampling conditions (such as swabbing the surface of harvested agricultural products [20]. The Raman spectra obtained using excitation lasers at 633 nm and 785 nm are shown in Figure 6a,c, respectively. In Figure 6c the spectra are offset for better visibility.

Distinctive Raman peaks corresponding to thiram molecules are usually observed at 400, 565, 980, 1146, 1370, 1490, and 2930 cm^−1^ [21].

Under 633 nm laser excitation, our AuNPs@BC SERS sensor enables the detection of the thiram band around 1380 cm^−1^ at concentrations as low as 2.4 ppm (Figure 6a). The band at around 560 cm^−1^ is barely distinguishable.

Under 785 nm excitation (Figure 6c), the 1370 cm^−1^ band remains detectable at concentrations as low as 0.24 ppm, and the band at 560 cm^−1^ becomes much more visible. Additionally, by comparing the spectra obtained with the two lasers, it can be observed that the spectra at 785 nm exhibit a significantly better signal-to-noise ratio than those at 633 nm. The AuNPs@BC displays cellulose characteristic peaks, in particular the band around 460 and 1090 cm^−1^ [22], which are especially noticeable with the 785 nm laser excitation (Figure 6c).

The spectra obtained with 633 nm excitation show a relatively uniform background of more than 1500 counts across the entire investigated range (see in particular 0 ppm spectrum in Figure 6a). This effect is drastically reduced at 785 nm, allowing for identifying and isolating the analyte peaks of interest (Figure 6c), along with additional peaks attributed to the cellulose itself.

The use of a 785 nm laser for Raman excitation in SERS experiments, even when the plasmon resonance of gold nanoparticles is around 500–530 nm, can be a better choice for several reasons.

One key reason is that longer wavelengths, such as 785 nm, help reduce background fluorescence from organic matrices like cellulose or biological samples. When shorter wavelengths, closer to visible light, are used, fluorescence becomes problematic, overpowering the weaker Raman signal [23].

Although the plasmon resonance of gold nanoparticles is strongest at shorter wavelengths, there is still some absorption and scattering at longer wavelengths, including 785 nm. This results in sufficient electromagnetic field enhancement to support the SERS effect, even if the enhancement is less intense than at the resonance peak [22].

Additionally, using a longer wavelength like 785 nm reduces the risk of overheating the nanoparticles. If the excitation wavelength were too close to the plasmonic resonance, excessive heating could cause nanoparticle aggregation or surface degradation, compromising both the stability and quality of the SERS measurement [23].

This is particularly problematic for biological samples, such as proteins or cells, which are highly sensitive to shorter wavelengths that can lead to photodegradation or excessive heating. Using a 785 nm laser significantly mitigates these risks, enabling non-destructive analysis of delicate biological materials. In summary, the 785 nm laser is the best choice to balance the need for signal amplification with practical considerations like minimizing background fluorescence, reducing sample damage, and ensuring the overall stability of the SERS system.

In addition to the previously discussed factors, the observed differences between the spectra excited at 633 nm and 785 nm also highlight the complementary nature of these wavelengths for SERS applications. While 633 nm excitation benefits from being closer to the plasmonic resonance of AuNPs, providing stronger enhancement, it is more prone to background fluorescence, particularly from the cellulose matrix. Conversely, 785 nm excitation significantly reduces this fluorescence, leading to a clearer baseline and better signal-to-noise ratio, as observed in our experiments. This trade-off allows 785 nm to detect lower concentrations of thiram (0.24 ppm) compared to 633 nm (2.4 ppm) for the characteristic 1370 cm^−1^ peak. The choice of excitation wavelength ultimately depends on the specific requirements of the analysis, such as sensitivity versus fluorescence suppression, underscoring the flexibility of the AuNPs@BC platform for diverse sensing scenarios.

To evaluate the sensitivity of the BC-based SERS substrate for thiram detection, SERS spectra were recorded by applying 5 μL of thiram standard solutions with concentrations ranging from 0 ppm to 2400 ppm. As the thiram concentration decreased, the intensity of the SERS spectra also diminished. Notably, the Raman peak at 1370 cm^−1^ remained detectable even at concentrations as low as 2.4 ppm under 633 nm excitation, and as low as 0.24 ppm under 785 nm excitation. A strong linear correlation (R^2^ = 0.99) was found between the average SERS intensities at 1370 cm^−1^ and the logarithm of the thiram concentrations (inset Figure 6b,d), indicating the potential for quantitative analysis across part of the tested concentration range. Therefore, based on the strong linear correlation, the thiram concentration in target samples can be accurately determined from the measured Raman intensity during the practical use of the AuNPs@BC SERS sensor.

## 4. Discussion

Our study highlights the potential of the AuNPs@BC platform as an eco-friendly and efficient substrate for SERS-based detection of thiram, achieving a detection limit of 0.24 ppm (1 µM). This result underscores the viability of using sustainable materials, such as bacterial cellulose, to create flexible, cost-effective, and disposable sensing systems tailored for agrifood applications. The unique architecture of the AuNPs@BC composite, combining the nanofibrillar structure of bacterial cellulose with the plasmonic properties of gold nanoparticles, enables both sensitivity and reproducibility. The refractometric and LSPR studies revealed a bulk sensitivity of 72 nm/RIU, highlighting the composite’s ability to detect small changes in the surrounding refractive index, which is essential for precise sensing applications.

The novelty of our work lies in the fabrication process of the composite film, where Au nanoparticles are in situ deposited onto the nano-fibrillated cellulose matrix, ensuring a uniform distribution of the nanoparticles. Unlike traditional methods that deposit AuNPs directly onto bacterial cellulose pellicles—often resulting in inhomogeneous nanoparticle distribution and rigid films upon drying—our approach maintains the flexibility even after drying without requiring the costly freeze-drying processes. The film thickness can be easily controlled by adjusting the amount of BC/AuNPs suspension during the filtration process, enabling production of scalable and customizable composite sheets.

When considering other approaches reported in the literature, it is evident that the detection capabilities depend significantly on the substrate design and analytical methodology. Techniques such as drop coating deposition Raman (DCDR) spectroscopy [24] and silver-based nanostructured SERS platforms [25] have demonstrated varying levels of sensitivity, often influenced by their specific experimental setups and substrate characteristics. While these systems may achieve extremely low detection limits, they frequently rely on more complex fabrication processes and rigid substrates, limiting their scalability and practical applicability.

In contrast, our approach prioritizes accessibility and environmental sustainability without compromising the detection limits required for practical applications, such as pesticide monitoring in food safety. By integrating gold nanoparticles into bacterial cellulose, we propose a platform that balances performance and practicality, supporting the development of scalable and disposable sensing devices for rapid and reliable field analyses.

Future developments could focus on optimizing the nanoparticle distribution within the cellulose matrix or exploring functionalization strategies to expand the range of detectable analytes. Additionally, integrating this platform into portable Raman systems could provide a robust tool for real-time field applications, offering significant advantages over existing technologies.

## 5. Conclusions

This study demonstrated the feasibility of gold nanoparticles (AuNPs)/bacterial cellulose (BC) composite for surface-enhanced Raman scattering (SERS) in the detection of agrochemical pesticides, specifically thiram. The nanofibrillar structure of the cellulose provided a high specific surface area and efficient dispersion of nanoparticles, creating a substrate with ideal electromagnetic hotspots for Raman signal amplification. The AuNPs@BC platform exhibited high reproducibility in its optical properties and sufficient sensitivity to detect thiram at concentrations below 1 ppm, suitable for detecting the lower concentration of pesticide residues according to safety regulatory.

The simplicity and sustainability of the production process make this substrate promising for the development of portable, cost-effective, and biodegradable sensors, with potential for in situ applications. Future studies may investigate the system’s capability for multiplexed analyte detection or apply the method to complex samples for quality control in the agri-food supply chain.

## Data Availability

Data are contained within the article.
